# The accuracy of soluble urokinase-type plasminogen activator receptor for the diagnosis of neonatal sepsis: a meta-analysis

**DOI:** 10.3389/fmed.2023.1169114

**Published:** 2023-04-27

**Authors:** Jinjin Ma, Xinqing Chen, Xiaoyan Wang, Jiaojiao Liang, Liyan Guo, Yan Su, Ling Hao, Changjun Ren

**Affiliations:** Department of Pediatrics, The First Hospital of Hebei Medical University, Shijiazhuang, Hebei, China

**Keywords:** neonatal sepsis, soluble urokinase-type plasminogen activator receptor, suPAR, diagnosis, meta-analysis

## Abstract

**Background:**

Neonatal sepsis is one of the major causes of morbidity and mortality in newborns. However, atypical clinical manifestations and symptoms make the early diagnosis of neonatal sepsis a challenge. Relatively high-serum soluble urokinase-type plasminogen activator receptor (suPAR) has been implicated as a diagnostic biomarker for adult sepsis. Therefore, the meta-analysis is intended to explore the diagnostic value of suPAR for neonatal sepsis.

**Methods:**

The PubMed, Cochrane Library, Embase, Web of Science, China National Knowledge Infrastructure, China Biological Medicine Disk, and Wanfang databases were retrieved from inception to 31 December 2022 to collect diagnostic accuracy studies about suPAR for neonatal sepsis. Two reviewers independently screened the literature, extracted data, and assessed the risk of bias in the included studies using the quality assessment of diagnostic accuracy studies-2 (QUADAS-2) tool. Then, a meta-analysis was performed using Stata 15.0 software.

**Results:**

A total of six articles involving eight studies were included. The results of the meta-analysis showed that the pooled sensitivity, specificity, positive likelihood ratio, negative likelihood ratio, and diagnostic odds ratio were 0.89 [95%CI (0.83–0.93)], 0.94 [95%CI (0.77–0.98)], 14 [95%CI (3.5–55.2)], 0.12 [95%CI (0.08–0.18)], and 117 [95%CI (24–567)], respectively. The area under the curve (AUC) of summary receiver operator characteristic (SROC) curves was 0.92 [95%CI (0.90–0.94)]. Sensitivity analysis confirmed the stability of the results, and publication bias was not observed. Fagan’s nomogram results demonstrated the clinical availability of the findings.

**Conclusion:**

Current evidence suggests that suPAR has potential diagnostic value for neonatal sepsis. Owing to the limited quality of the included studies, more high-quality studies are needed to verify the above conclusion.

## 1. Introduction

While neonatal care has evolved over the years and neonatal sepsis-related deaths have fallen, it remains an important cause of mortality for neonates, especially for preterm infants (1). The incidence of septic shock has been reported to reach 1.3% among patients in neonatal intensive care units (NICUs) (2). If early diagnosis and appropriate treatment are not made in time, it is very easy for organ failure to occur in children, and it is even a fatal threat to neonates. Although blood culture is considered the gold standard for diagnosing sepsis, this process cannot produce immediate results, and a large blood sample is required to provide optional results (3). Common biomarkers, such as C-reactive protein (CRP), procalcitonin (PCT), and interleukin-6 (IL-6), are generally associated with inflammation, and thus, their specificity for infection is low and could be affected by many other reasons (4). Consequently, challenges remain in achieving early recognition, accurate diagnosis, and standardized management of neonatal sepsis.

Currently, various biomarkers, biological molecules that are characteristic of normal or pathogenic processes and can be easily and objectively measured, have been proposed as being of potential use for sepsis diagnosis, therapeutic guidance, and/or prognostication (5, 6). Urokinase-type plasminogen activator receptor (uPAR) is a single-strand transmembrane glycoprotein that is expressed in neutrophils, monocytes macrophages, T cells, dendritic cells, and other inflammatory and immune cells. The natural immune response and the inflammatory process can be affected by uPAR through its influence on chemotaxis and phagocytosis of pathogens, and its interaction with extracellular matrix components, such as vitronectin and integrins. After being cleaved and released from the cell membrane, uPAR is recognized as a soluble receptor (7) that can be found in various bodily fluids, including blood, urine, cerebrospinal fluid, and saliva. Back in 1995, elevated plasma suPAR levels were reported in a small group of septic intensive care unit (ICU) patients (8). Since then, a growing body of evidence has shown that suPAR blood levels increase in conditions with severe inflammation and immune activation, such as infectious, autoimmune, and neoplastic diseases (9). Additionally, suPAR appears to discriminate better than some other biomarkers among patients with different severities of illness (10). Recently, numerous studies have shown that an early increase in suPAR levels predicts severe respiratory failure (11), acute kidney injury (12), and death in patients with coronavirus disease 2019 (COVID-19). Based on these findings, Kyriazopoulou et al. designed the suPAR-guided Anakinra treatment for Validation of the risk and Early Management Of severe respiratory failure by COVID-19 (SAVE-MORE) study to evaluate the efficacy and safety of early initiation of anakinra treatment in hospitalized patients with moderate or severe COVID-19 (13). This study was approved by the US emergency use authorization (EUA) and the food and drug administration (FDA). As a long-term inflammatory biomarker, suPAR has attracted widespread attention.

The diagnostic value of suPAR in neonatal sepsis has been reported in the literature; however, there are significant variations among different studies. The present meta-analysis aims to explore the accuracy of suPAR in diagnosing neonatal sepsis and provide evidence-based support for whether suPAR can be used as an early diagnostic marker of neonatal sepsis.

## 2. Materials and methods

### 2.1. Search strategy

Our meta-analysis was designed according to the preferred reporting items for systematic reviews and meta-analyses guidelines for diagnostic test accuracy (PRISMA-DTA), which are shown in the Supplementary material. We retrieved the PubMed, Cochrane Library, Embase, Web of Science, China National Knowledge Infrastructure (CNKI), China Biological Medicine Disk (CBM), and Wanfang Data databases using the search terms ‘suPAR’, ‘Urokinase Plasminogen Activator Receptor’, ‘soluble’, ‘newborn’, ‘premature infant’, ‘sepsis’, ‘neonatal sepsis’, and so on. To enhance the recall and precision ratio, we used the combination of medical subject headings (MeSH) and entry terms mainly in our primary search. The date of our last search was set at 31 December 2022.

### 2.2. Study selection

Two researchers (JM and XC) independently selected the literature and cross-checked and negotiated with a third party (LH) in case of differences. The following criteria were applied to identify studies for inclusion in our meta-analysis: (1) original report published on the accuracy of suPAR in diagnosing neonatal sepsis, including case-control and cohort studies; (2) the researchers were able to extract information from the 2 × 2 contingency table so that true positive (TP), true negative (TN), false positive (FP), and false negative (FN) values could be directly or indirectly obtained; (3) the subjects were newborns within 28 days after birth; and (4) without language restrictions. The exclusion criteria were as follows: (1) the outcome was inconsistent with the criteria of neonatal sepsis; (2) duplicate publication; (3) conference papers, abstracts, and lectures; and (4) literature unable to extract data of diagnostic four grid table.

### 2.3. Definitions

Neonatal sepsis definitions do not align with those used for adults and children, as many clinicians still rely on microbiological results rather than organ dysfunction (14). The signs and symptoms of sepsis are non-specific and include temperature instability (usually with fever), irritability, lethargy, tachypnea, grunting, hypoxia, poor feeding, tachycardia, poor perfusion, and hypotension (15). Clinical sepsis is defined as the presence of at least two typical clinical signs along with two laboratory abnormalities. Culture-proven sepsis requires a positive microbial blood culture.

Overall, clinicians need to be aware of the differences in sepsis definitions in neonates and the challenges of diagnosing sepsis based on non-specific symptoms. A more comprehensive approach, including both clinical and laboratory findings, can help accurately identify neonatal sepsis and guide appropriate treatment.

### 2.4. Data extraction

Based on the preset inclusion and exclusion criteria, the data we extracted mainly included the first author’s name, publication year, country, type of sample, gestation age, birth weight, sepsis onset, reference standard, sample size, TP, FP, FN, and TN.

### 2.5. Quality assessment

The quality assessment of diagnostic accuracy studies-2 (QUADAS-2) tool was used to assess the quality of the selected studies. This test comprises four key domains that discuss patient selection, index test, reference standard, and flow of patients through the study, as well as the timing of the index tests and reference standard (flow and timing) (16). Two review authors (XW and JL) individually conducted the assessment and cross-checked each other’s work. Any disagreements were resolved through discussion or by seeking the opinion of a third author (CR) to reach a consensus.

### 2.6. Statistical analysis

All analyses were undertaken using Stata 15.0 statistical software. Hierarchical summary receiver operating curves (HSROCs) along with Spearman’s correlation coefficient were used for estimating the heterogeneity caused by the threshold effect. Furthermore, the statistical heterogeneity among the research results was analyzed by Cochran Q statistic, and the *I*^2^-test was used to quantitatively judge the size of heterogeneity. When study heterogeneity was statistically significant (*I*^2^ ≥ 50% or *p* ≤ 0.05), the random effect model was used; otherwise, a fixed effects model was used (17). The obvious heterogeneity was treated by sensitivity analysis. To evaluate suPAR potential and accuracy in neonatal sepsis diagnosis, sensitivity (Sen), specificity (Spe), positive likelihood ratio (PLR), negative likelihood ratio (NLR), diagnostic odds ratio (DOR), pretest probability, post-test probability, and area under the curve (AUC) of summary receiver operating characteristic (SROC) were used. Deeks’ funnel plot asymmetry test was used to evaluate publication bias (18).

## 3. Results

### 3.1. Study characteristics

**Figure 1:**
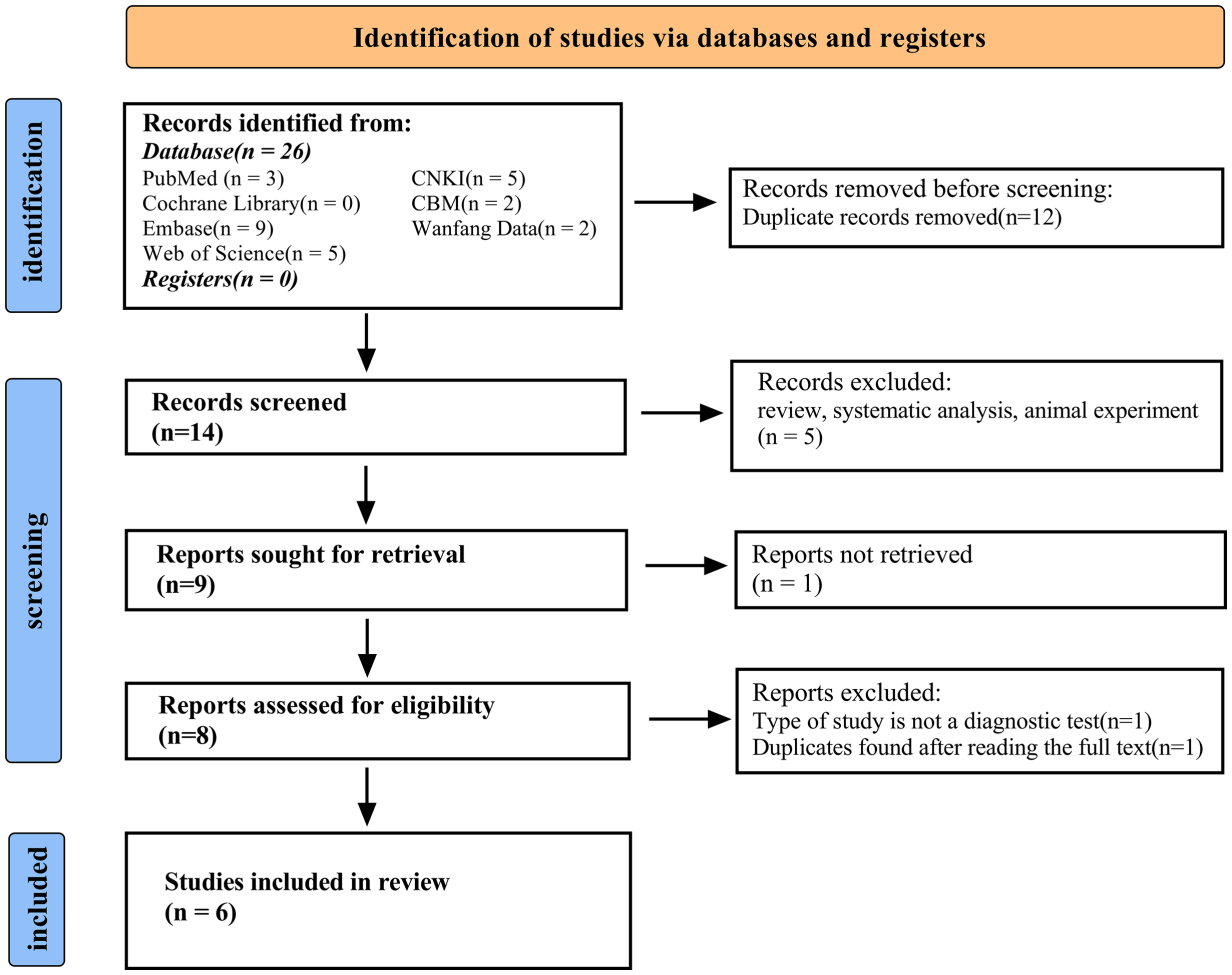
PRISMA (preferred reporting items for systematic reviews and meta-analyses) flow diagram and exclusion criteria.

After a preliminary search, a total of 26 studies were identified, and 17 of them were weeded out by our exclusion criteria. Reading the title, abstract, and full text, the remaining six articles were included in our study (7, 19–23). Figure 1 shows the selection progress. The six articles, including eight studies, involved 212 neonates with sepsis, of which 141 explicitly mentioned their blood culture was positive. They also included 161 infected neonates without sepsis and 231 healthy neonates. The characteristics of the six articles incorporated into our study are displayed in Table 1.

**Table 1:** Characteristics of the included studies.

Author and year	Country	Type of sample	Gestation age(w)	Birth weight(g)	Sepsis onset	Reference standard	Sample size	TP	FP	FN	TN	Threshold (ng/ml)
Cekmez (19)	Turkey	Neonate	P 36.1 ± 2.7	2,420 ± 368	Mixed	Culture-proven sepsis	60	38	1	2	19	13.63
			C 36.0 ± 2.3	2,520 ± 280								
Okulu (21)	Turkey	Neonate	P 31.8 ± 4.2	1689.6 ± 914.5	Late	At least 3 sepsis-related clinical signs	66	8	0	2	56	11.3
						CRP > 1 mg per 100 ml						
			C 33 ± 2.4	1983.8 ± 535.5		At least two other altered serum parameters in addition to CRP						
						blood culture; positive or negative						
Siahanidou (7)	German	Term	P 39 ± 1.0	3,135 ± 351	Mixed	Culture-proven sepsis	65	8	5	5	47	4.79
			C 38.6 ± 1.0	3,216 ± 406								
Li (22)	China	Preterm	P 31.9 ± 0.5	1904 ± 48	Late	Culture-proven sepsis	85	36	0	4	45	10.9
			C 32.4 ± 0.2	2048 ± 61		Clinical sepsis						
Fu (23)	China	Term	P 37 ~ 42周	-	Early	Culture-proven sepsis	438	28	275	3	132	12.01
			C-			Clinical sepsis		25	69	6	338	suPAR 12.01 sICAM 349.50
Niu (20)	China	Neonate	P 32.5 ± 10.0	1906.3 ± 248.9	Mixed	Culture-proven sepsis	150	68	9	7	66	10.76
			C 31.2 ± 9.8	2107.0 ± 298.4				70	3	5	7	hs-CRP 10 m/l suPAR 10.76

The ‘-’ means that this term is not mentioned in the article.

P, patients in the case groups; C, control group; TP, the patients’ number of reference standard positives with a positive index test; FP, the patients’ number of reference standard negatives with a positive index test; FN, the patients’ number of reference standard positives with a negative index test; TN, the patients’ number of reference standard negatives with a negative index test.

### 3.2. Quality assessment

**Figure 2:**
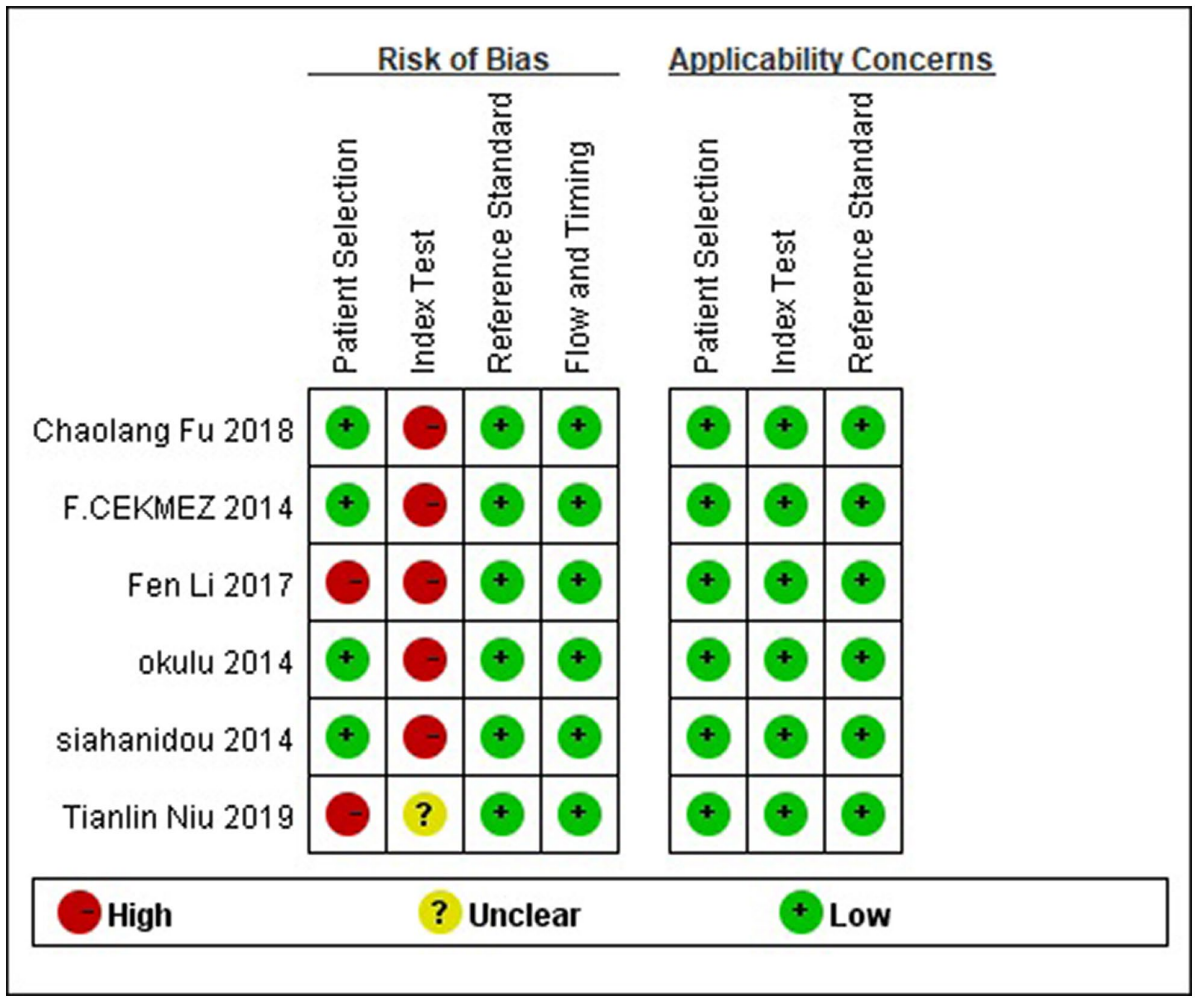
Quality assessment of diagnostic accuracy studies-2 (QUADAS-2) tool.

The risk of bias and the applicability of the included study were assessed using QUADAS-2. The outcomes are illustrated in Figure 2. Four studies (7, 19, 21, 23) used a prospective study design that avoided inappropriate exclusion. The remaining articles with case–control design may exaggerate diagnostic accuracy. As shown, the high risk of bias was mainly detected in the domain of the index test since the included studies did not use pre-specified thresholds but the optimal ones on the ROC curve in their analysis.

### 3.3. Heterogeneity analysis and diagnostic accuracy

**Figure 3:**
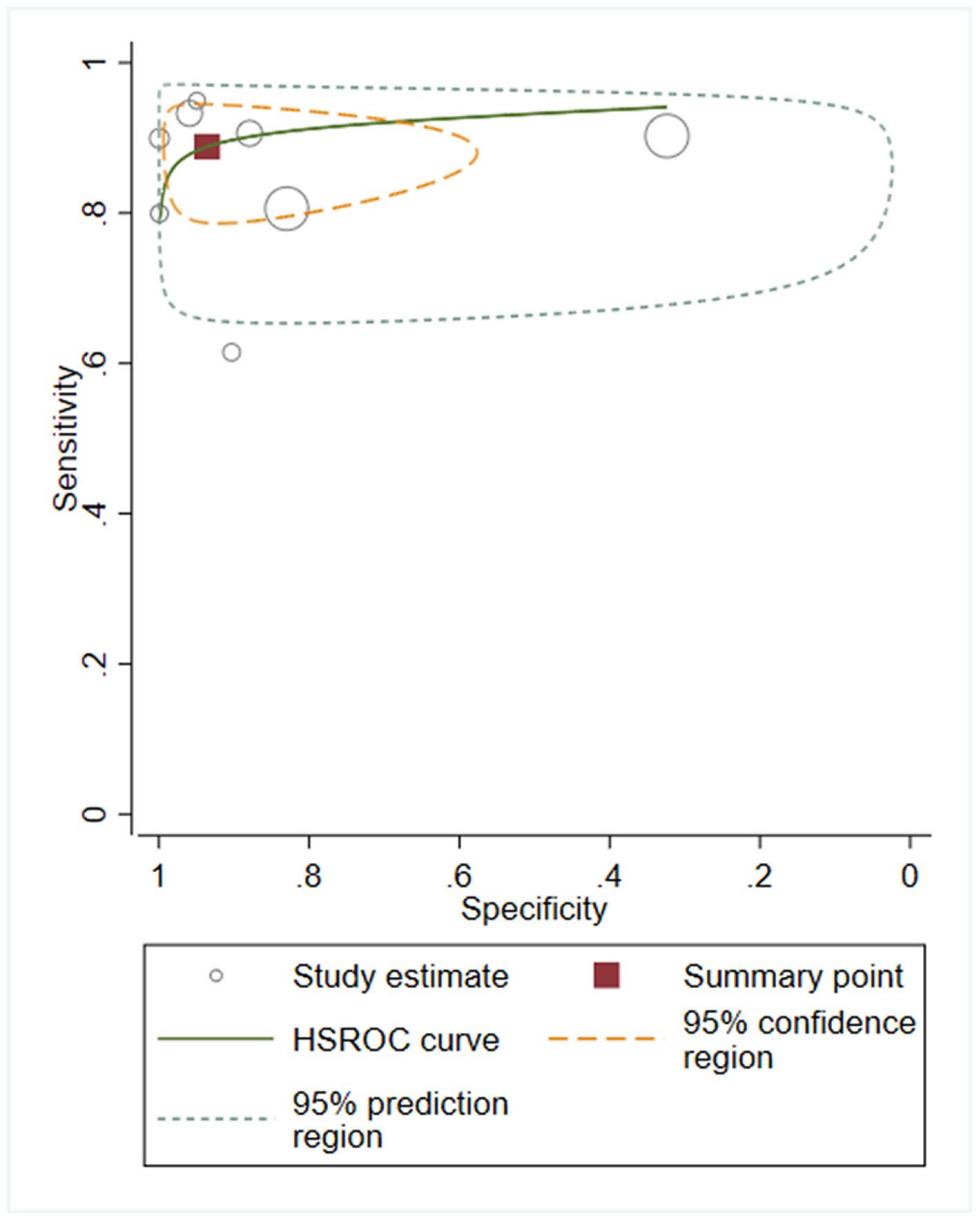
Hierarchical summary receiver operator characteristic (SROC) curve.

**Figure 4:**
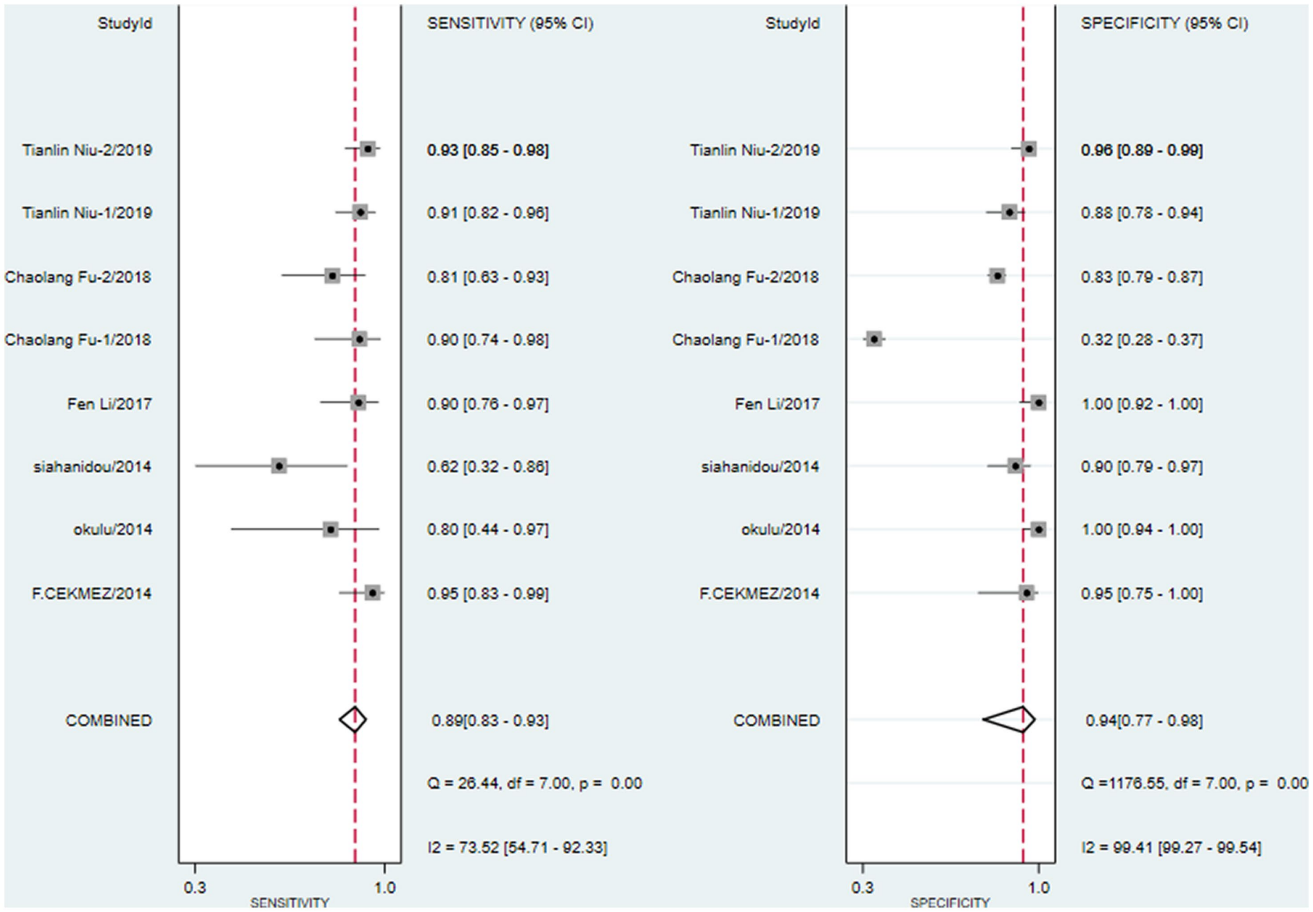
Forest plots for pooled sensitivity and specificity of neonatal sepsis diagnosis by soluble Urokinase-type plasminogen activator receptor (suPAR).

**Figure 5:**
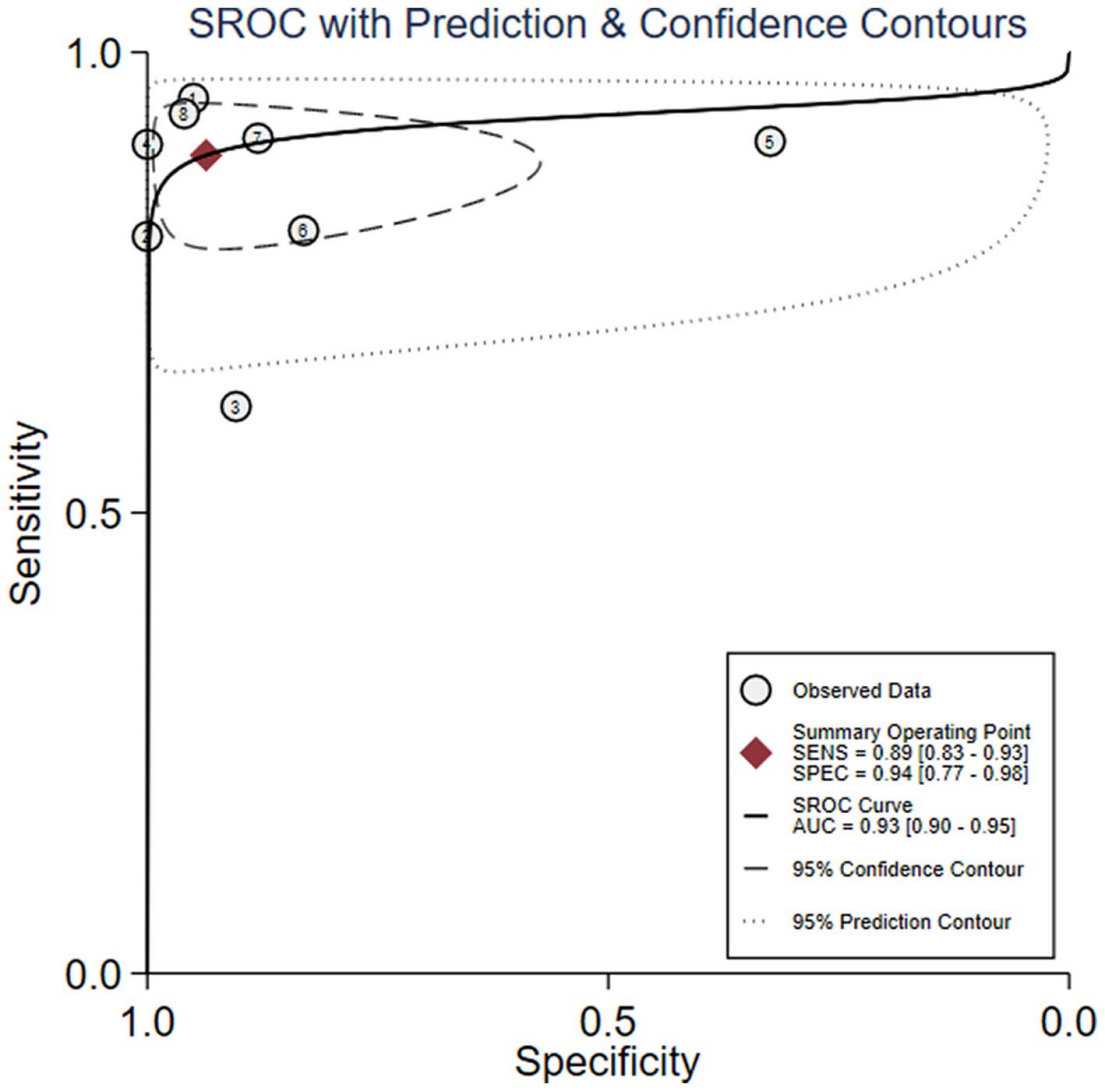
Soluble Urokinase-type plasminogen activator receptor (suPAR) symmetrical summary receiver operator characteristic (SROC) curve for all eight studies.

We first performed heterogeneity analysis by using the HSROC model to estimate, which is shown in Figure 3. Spearman’s correlation analysis of sensitivity and (1-specificity) logarithm showed that the correlation coefficient was 0.314 (*p* = 0.544), indicating that there was no threshold effect. However, the sensitivity and specificity of I^2^ were above 50%, which means there was heterogeneity between studies so the random effect model was used for statistical analysis. The results are displayed in Figure 4. As it turns out, the overall sensitivity and specificity of the eight studies were 0.89 [95%CI (0.83–0.93)] and 0.94 [95%CI (0.77–0.98)], respectively. The PLR was 14 [95%CI (3.5–55.2)], the NLR was 0.12 [95%CI (0.08–0.18)], and the DOR was 117 [95%CI (24–567)]. The SROC curve analysis of suPAR test accuracy in neonatal sepsis diagnosis revealed an AUC of 0.92 [95%CI (0.90–0.94)] (Figure 5).

### 3.4. Sensitivity analysis

**Figure 6:**
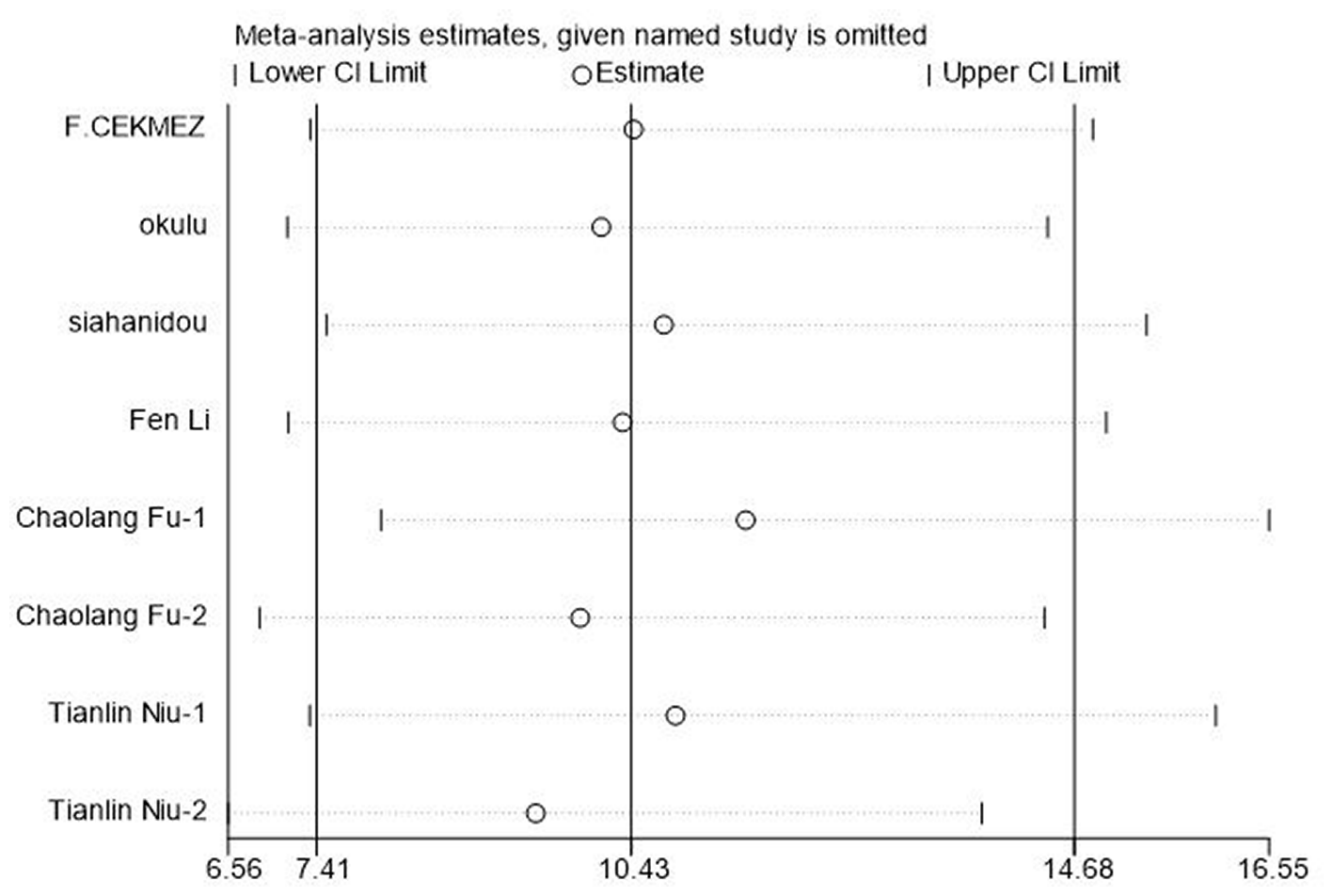
Sensitivity analysis of soluble Urokinase-type plasminogen activator receptor (suPAR) for neonatal sepsis diagnosis.

To evaluate the reliability and robustness of the analysis results, we rejected individual studies in turn and remerged with the remaining research. The result showed that it has little impact on the amount of merger effect regardless of which study has been excluded (Figure 6). In other words, our research results are relatively stable, and the analysis results are highly reliable.

### 3.5. Clinical utility of the index test

**Figure 7:**
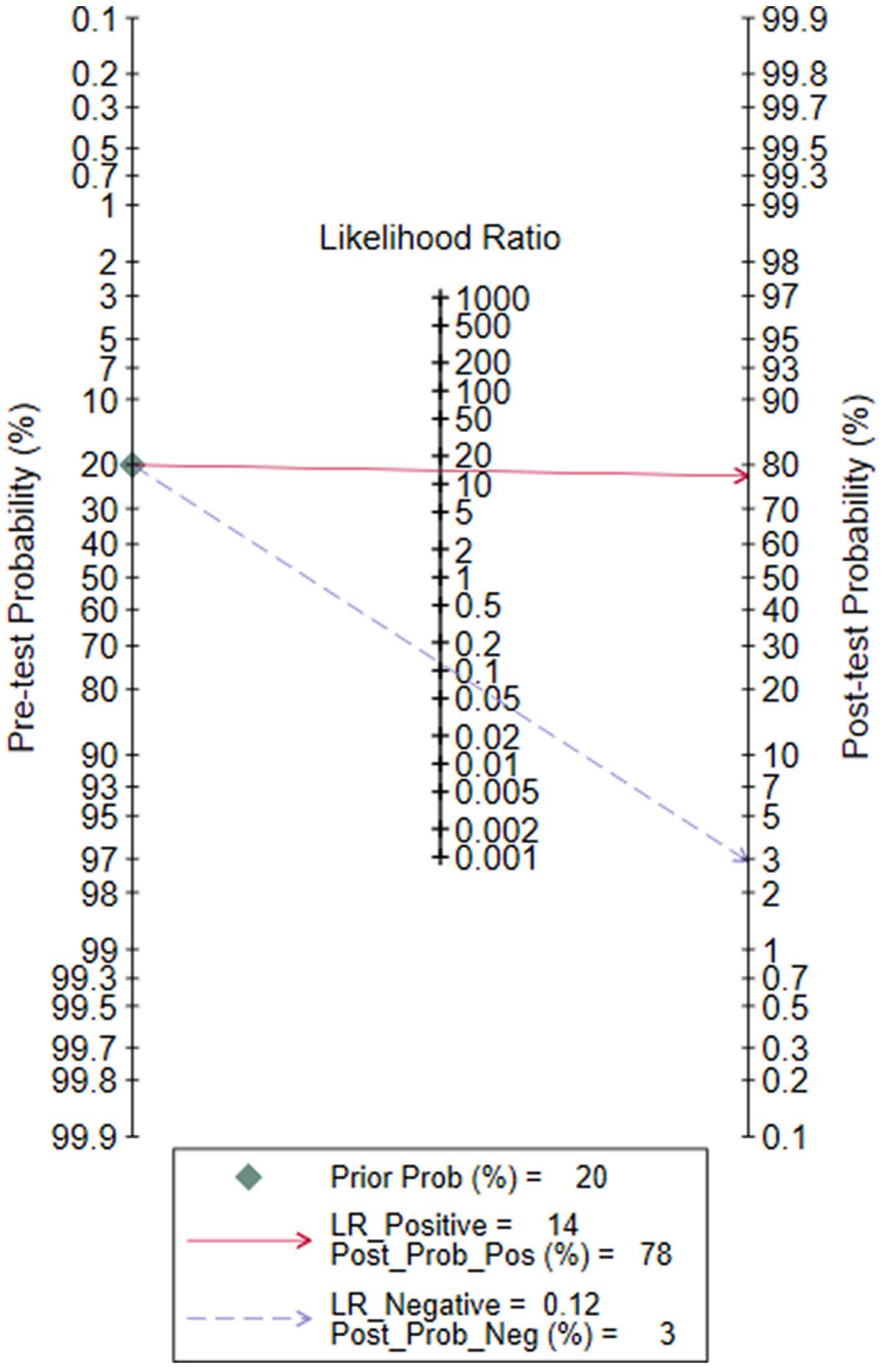
Fagan’s nomogram for calculating the post-test probabilities of soluble Urokinase-type plasminogen activator receptor (suPAR) for neonatal sepsis diagnosis.

The Fagan graph was plotted to find out valuable clinical utility. Fagan’s nomogram indicated that, if the result of a diagnostic test was positive, the probability that the neonates suffered sepsis would increase from the pretest risk of 20 to 78%. If the result was negative, the probability that the newborn was affected with sepsis decreased from a pretest risk of 20 to 3% (Figure 7).

### 3.6. Publication bias

**Figure 8:**
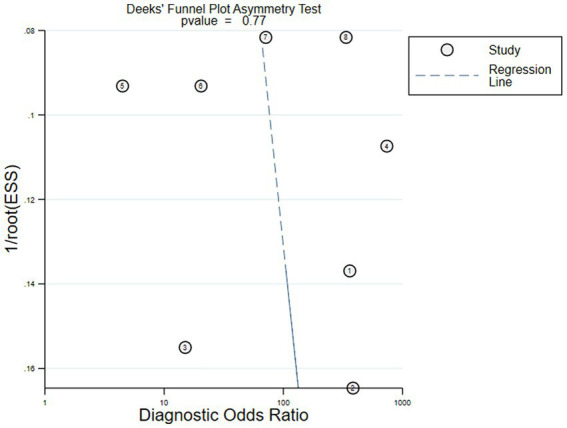
Deeks’ funnel plot for identifying publication bias.

The Deeks’ funnel plot asymmetry test on the six included studies showed that there was no obvious asymmetry (*p* = 0.77), and it indicated that the pooled results were not influenced by the publication bias if the value was 0.05 as a standard test (Figure 8).

## 4. Discussion

The soluble form of uPAR, known as suPAR, maintains a stable serum level regardless of harvesting time, diet (24), biological circadian rhythm (25), and repeated freezing dissolution. In recent years, suPAR has been shown to play important regulatory roles in various immunological functions and has been extensively studied as a modern inflammation marker. Several observational studies have suggested that the increased serum levels of suPAR are associated with a variety of systemic inflammatory disorders, such as infection by the human immunodeficiency virus-1 and diffuse carcinomatosis (26). A meta-analysis by Huang et al. (27) on adult sepsis has proved that suPAR has moderate diagnosis and prognosis value.

Nevertheless, these results most certainly cannot be directly extrapolated to neonatal patients with sepsis due to the widely different conditions, age, developmental stage, and overall state of the organism struck by neonatal sepsis (28). To the best of our knowledge, this present meta-analysis is the first reported investigation of the diagnostic value of suPAR in neonatal sepsis.

Our results indicated that detecting suPAR in neonatal sepsis had high sensitivity and specificity. The pooled data from eight studies showed that the AUC of suPAR in diagnostic value was 0.92. Currently, C-reactive protein (CRP) is the most studied biomarker (29), and in a recent meta-analysis of CRP for neonatal sepsis (30), the pooled sensitivity was 0.74 and specificity was 0.62. A systematic review of 1,959 patients reported that the sensitivity and specificity of PCT were 81% [95%CI (74–87%)] and 79% [95% CI (69–87%)], respectively (31). Compared with the previous meta-analysis, our results found that suPAR exhibited higher specificity than CRP and PCT, suggesting that it can better identify non-sepsis neonates and is more distinguishable than other biomarkers in newborns with different inflammatory diseases. The DOR of this research is 117, indicating that suPAR has high diagnostic efficiency by combining results from different studies into summary estimates with increased precision (32). In this study, positive and negative likelihood ratios were also selected as measurement indicators of diagnostic efficiency. With a PLR and NLR of 14 and 0.12, respectively, on the one hand, the positive rate of suPAR in neonates with sepsis is 14 times higher than without sepsis, and cases of suPAR showing positive should accept further examination to confirm the diagnosis. On the other hand, if suPAR is negative, the probability of neonatal sepsis is 12%, which means it has a good elimination effect. Considering all these results, it appears that suPAR has outstanding accuracy in diagnosing neonatal sepsis. However, whether suPAR can be used as a final diagnostic index is still inconclusive. Furthermore, a few studies have suggested that high suPAR plasma levels closely correlate with morbidity and mortality in septic patients, demonstrating its value as a prognostic biomarker in systemic inflammation and sepsis (33, 34). One of the studies we included also confirmed that the level of first-day suPAR can help predict the prognosis of neonatal sepsis (22).

As the forest plot illustrates, there is some heterogeneity in the estimates of sensitivity and specificity, which may reduce the robustness of the results to some extent. At present, blood culture is still the ‘gold standard’ for the diagnosis of neonatal sepsis, but its positive rate is low due to factors such as blood collection, culture conditions, and antibiotic treatment. As a result, most sepsis diagnoses in the included studies were based on clinical diagnosis, with three studies clearly stating that positive blood culture was used as the standard for sepsis inclusion. However, there was not enough data to support the subgroup analysis of blood culture-positive and clinically diagnosed sepsis. Fortunately, the threshold effect, as a potential influencing factor, was found not to exist. Beyond that, other factors that may cause heterogeneity include gestational age, birth weight, blood sample processing conditions, and suPAR detection methods. However, the included studies did not provide sufficient data to explore the potential association of these factors. Therefore, to ensure the highest possible accuracy for the diagnostic efficiency of suPAR, prospective studies with rational design, high quality, large sample sizes, and long-term follow-up should be considered as much as possible.

The diagnostic threshold plays a crucial role in disease diagnosis. Despite the increasing number of studies on the diagnosis of neonatal sepsis by suPAR, the normal range of its serum has not yet been determined. Small sample studies show that the adult blood suPAR level is 1.2–4.0 ng/ml, while the newborn blood suPAR level is 3.7–10.8 ng/ml (7, 21, 35, 36). The critical level of plasma suPAR in the six works of literature included in this meta-analysis was 4.79–13.63 ng/ml, which is consistent with previous research both domestically and internationally. However, except for Niu et al.’s study, none of the studies included in this meta-analysis predetermined the diagnostic threshold, which may have a certain impact on the results. Therefore, future research should pay attention to exploring the correlation between suPAR and neonatal sepsis and determining its optimal critical value. Although this study is heterogeneous, it still provides a valuable reference for future research.

There are several limitations to the current meta-analysis. First, the limited number of included studies may have an impact on the result of the meta-analysis. Second, our review did not investigate the diagnostic value of suPAR when used in conjunction with other biomarkers. Third, it was difficult to obtain the raw data for each included study, which restricts us to explore the prognosis value of suPAR in neonatal sepsis. More importantly, owing to the uniqueness of neonatal infection, the relationship between the cutoff level of suPAR and age after birth in full-term and preterm infants needs to be further studied. Finally, sources of heterogeneity in the results should still be considered carefully. Nevertheless, no significant publication bias was found in this study, and the sensitivity analysis results did not change significantly, indicating that the research conclusions are reliable to a certain extent.

## 5. Conclusion

The existing evidence shows that suPAR has a high diagnostic value for neonatal sepsis and has a certain clinical guiding role in reducing neonatal sepsis mortality. In clinical application, symptomatic newborns who test negative for suPAR cannot be ruled out for neonatal sepsis. Clinical practice is needed to determine whether symptomatic newborns who test positive for suPAR have neonatal sepsis. Based on the existing research defects, more prospective studies with reasonable design and long-term follow-up are needed to clarify the diagnostic value of suPAR in neonatal sepsis.

## Data availability statement

The original contributions presented in the study are included in the article/Supplementary material, further inquiries can be directed to the corresponding authors.

## Author contributions

JM and XC planned and designed the direction of this study. They are also the original drafters of this paper. XW, JL, JM, and XC participated in retrieving the literature. LG and YS collected the data. JM, XC, XW, and LH implemented the data processing and analyzing. In the end, JM, XC, LH, and CR completed the final reviewing and editing. All authors contributed to the article and approved the submitted version.

## Funding

This study was supported by Hebei Province Science and Technology Support Program, China under Grant No. 21377738D.

## Conflict of interest

The authors declare that the research was conducted in the absence of any commercial or financial relationships that could be construed as a potential conflict of interest.

## Publisher’s note

All claims expressed in this article are solely those of the authors and do not necessarily represent those of their affiliated organizations, or those of the publisher, the editors and the reviewers. Any product that may be evaluated in this article, or claim that may be made by its manufacturer, is not guaranteed or endorsed by the publisher.

## Supplementary material

The Supplementary material for this article can be found online at:

https://www.frontiersin.org/articles/10.3389/fmed.2023.1169114/full#supplementary-material
